# Factors Associated with Depressive Symptoms among Mexican-Origin Adults in a Community Sample at the US Mexico Border Region

**DOI:** 10.3390/ijerph20116017

**Published:** 2023-05-31

**Authors:** Mario Morales, Maia Ingram, Kiera M. Coulter, Thomas Nuño, Ada M. Wilkinson-Lee, Jill E. Guernsey De Zapien, Scott Carvajal

**Affiliations:** 1 Arizona Prevention Research Center, Health Promotion Sciences Department, Mel & Enid Zuckerman College of Public Health, The University of Arizona, Tucson, AZ 85724, USA; 2 Population Research Center, College of Liberal Arts, The University of Texas, Austin, TX 78712, USA; 3 Department of Mexican American Studies, The University of Arizona, Tucson, AZ 85724, USA

**Keywords:** depressive symptoms, Mexican-origin adults, social determinants of health, US–Mexico border

## Abstract

Using baseline data from three partnering federally qualified health centers, we examined factors associated with depressive symptoms among Mexican-origin adults at risk of chronic disease living in three counties in Southern Arizona (i.e., Pima, Yuma, and Santa Cruz). Multivariable linear regression models identified correlates of depressive symptoms for this population controlling for sociodemographic characteristics. Among 206 participants, 85.9% were female and 49% were between 45 and 64 years of age. The proportion of depressive symptoms was 26.8%. Low levels of physical pain and high levels of hope and social support were also reported. Physical pain was positively and significantly related to depressive symptoms (β = 0.22; 95% CI = 0.13, 0.30). Conversely, hope was negatively and significantly associated with depressive symptoms (β = −0.53; 95% CI = −0.78, −0.29). A better understanding of factors related to depressive symptoms among Mexican-origin adults is necessary to fulfill their mental health needs, as well as to achieve health equity and to eliminate health disparities in the US–Mexico border region.

## 1. Introduction

Mexican-origin people are the largest sub-group of Latine in the US (~66% out of 58.9 million in 2017) [1]. Depression disorders and depressive symptoms, such as the diagnosed and undiagnosed presence of sad, empty, or irritable mood, accompanied by semantic and cognitive changes [2], are one of the most common mental health problems among Mexican-origin adults [3]. Additionally, their reported levels of depressive symptoms are higher compared to non-Latine adults (~16% to ~40% vs. ~9%) [3,4]. In comparison to whites, Mexican-origin older men are also more likely to report having untreated past-year clinical depression [5,6]. However, relatively few studies have examined either diagnosed depression and depressive symptoms along the US–Mexico border region [3,5,7,8]. These studies have shown that depressive symptoms are positively associated with being female, lower educational attainment, unemployment, morbid obesity, diabetes, anxiety symptoms, HIV infection, cross border ties, childhood sexual abuse, substance use, and high-risk sex. Conversely, depressive symptoms have been negatively related to high self-rated health status, self-esteem, physical activity, high diet quality, and neighborhood collective efficacy. Controlling for sociodemographic characteristics, the current study complements prior research by focusing on two potential positive factors (i.e., hope, and social support), and two potential negative factors (i.e., physical problems severity and anxiety symptoms) that may be associated with depressive symptoms among Mexican-origin adults living in three Arizona–Sonora border counties. COVID-19 greatly aggravated mental health vulnerabilities among individuals living in the US–Mexico border region [9], increasing the need for research on factors related to mental health and depressive symptoms in the region. This study will inform ongoing and future surveillance efforts as well as interventions on depressive symptoms in the US–Mexico border region.

### 1.1. Potential Positive Factors Correlated to Depressive Symptoms

Hope, or positive expectations for the future [10,11], has been shown to be protective against depressive symptoms [12,13]. Among Mexican-origin females in Arizona, depressive symptoms were positively and significantly associated with hopelessness [11]. Social support, such as having individuals available to assist (e.g., love and care) when needed [8], has been known to be negatively correlated with depressive symptoms among Latine [14]. Specifically, among Mexican migrant farmworkers in northwest Ohio/southeast Michigan, social support was negatively related with depressive symptoms [15].

### 1.2. Potential Negative Factors Correlated to Depressive Symptoms

Prior studies have found a significant association between Latine ethnicity and physical pain reports, as well as an association between depressive symptoms and pain reports (as the number of physical problems increases, so do the likelihood of depressive symptoms) [16]. Particularly, in a mental health clinic in Starr County, Texas, Mexican-origin patients meeting the criteria for a major depressive episode were more likely to have one or more pain complaints than those not meeting the criteria [17]. Anxiety, a future-oriented mood state (e.g., worry and avoidance) in preparation for negative events [18], has also been highly correlated with depressive symptoms among Latine individuals [13]. Explicitly, depressive symptoms and pregnancy-related anxiety symptoms were associated among both Mexican American and Mexican immigrant females [19].

Controlling for sociodemographic characteristics, this study aimed to examine whether hope, social support, physical pain, and anxiety symptoms were statistically and significantly associated with depressive symptoms among Mexican-origin adults from a community sample within three border counties in Southern Arizona. It hypothesized that hope and social support would be negatively related to depressive symptoms, and that the severity of physical problems and anxiety symptoms would be positively associated with depressive symptoms. Studies on mental health in the US–Mexico border region are particularly relevant considering the structural constraints that prevail: high rates of poverty, under-education, unemployment, comorbid chronic conditions, racial discrimination, lack of health insurance coverage, and paucity of healthcare professionals [5]. This study provides insight to the importance of increased access, acceptability, and availability of mental health resources in a medically underserved region.

## 2. Materials and Methods

### 2.1. Study and Intervention

This study was undertaken as part of the Linking Individuals Needs to Community and Clinical Services (LINKS) project, a prospective matched observational study design with electronic health records (EHR), which has been described elsewhere [20]. LINKS was a study in which the clinic-based Community Health Workers (CHW) recruited eligible participants (i.e., word of mouth, clinic waiting rooms, health fairs, and other community settings) and referred them (i.e., by phone, REDCap, or in-person) to the community-based CHW, who then met with the participants in three federally qualified health centers to complete the baseline survey either in English or Spanish (July 2017 to September 2018). Community-based CHW also identified participants’ needs, referred them to programs and services, and taught them emotional well-being techniques [20]. Federally qualified health centers represent the back-bound primary care services to uninsured and underinsured populations in the US; the partners are either the sole or largest of such providers in the counties of the study.

### 2.2. Sample and Setting

As described in the LINKS protocol [20], this is a non-randomized study. However, a total of 250 participants would provide 90% power to detect a between group difference of 0.3 standardized units using a 4:1 allocation of controls to LINKS participants at a two-sided α = 0.05/8 significance level. The sample consisted of 213 Mexican-origin adults who completed baseline assessments. Inclusion criteria were as follows: age ≥ 21, being a English or Spanish speaker, and being at risk of or with a chronic disease (according to individuals’ EHRs on BMI, HbA1C, blood pressure, and lipids) [20]. CHWs obtained written consent from all LINKS participants. The University of Arizona Institutional Review Board approved all stages of the research (1612044741R001).

### 2.3. Questionnaire

The LINKS project was part of a community-based participatory study developed between an academic institution and a group of community partners that included health centers, county health departments, and other community health representatives. The partners worked collaboratively in all aspects of the study, including developing the Emotional Well-being Questionnaire. This questionnaire included a depressive symptom measure adapted from the Center for Epidemiologic Studies Depression Scale (CES-D-R 10; [7]). Other instruments included adaptions (for language and local responsiveness) of the State Hope Scale (SHS; [21]), the Short Form 8 Health Survey (SF-8; [22]), the Social Support Inventory—Enhancing Recovery in Coronary Heart Disease (SSI-ENRICHED; [23]), and sociodemographics [20]. Questionnaires that specifically target sub-threshold levels of symptoms, which do not meet the diagnostic criteria, hold significance because individuals experiencing subthreshold depressive symptoms or minor depression still face impairment, a heightened risk of developing major depression, and are prone to suicidal behaviors [24].

### 2.4. Measurements

Depressive symptoms (adapted version of the Center for Epidemiologic Studies Depression Scale (CESD)) were measured using seven items asking about negative affect, somatic complaints, or social isolation experienced during the past four weeks (i.e., “I was bothered by things that usually don’t bother me”, “I had trouble keeping my mind on what I was doing”, “I felt depressed”, “I felt that everything I did was an effort”, “I felt fearful”, “I felt lonely”, and “I could not get going”). Response categories scored 1 = rarely/none of the time, 2 = some/little of the time, 3 = occasionally/moderate amount of time, and 4 = all the time. Participants’ responses were summed to generate a score from 7 to 28, with higher scores indicating more severe depressive symptoms. The reliability coefficient for this sample was good (Cronbach’s α = 0.851; 95% CI: 0.796, 0.887). The questionnaire also included the following question: Do you have any of the following health problems? Depression (No/Yes), which was used to provide a binary estimate of the proportion of depressive symptoms in the sample.

Hope (State Hope Scale, SHS). Community partners identified hope as an important factor in emotional wellness. Hope was measured using six items asking about current agency and pathways to reach goals (i.e., “If I should find myself in a jam, I could think of many ways to get out of it”, “At the present time, I am energetically pursuing my goals”, “There are lots of ways around any problem that I am facing now”, “Right now I see myself as being pretty successful”, “I can think of many ways to reach my current goals”, and “At this time, I am meeting the goals that I have set for myself”). Answer options were reversed: 1 = none of the time, 2 = a little of the time, 3 = some of the time, 4 = most of the time, and 5 = all the time. Summed scores ranged from 9 to 30, with higher scores representing a higher expression of hope. The reliability coefficient for this sample was good (Cronbach’s α = 0.879; 95% CI: 0.846, 0.904).

Social support Inventory (Enriching Recovery in Coronary Heart Disease (ENRICHED)). Six items addressed perceptions of social support during the past four weeks (i.e., “Is there someone available to you whom you can count on to listen to you when you need to talk?”, “Is there someone available to give you good advice about a problem?”, “Is there someone available to you who shows you love and affection?”, “Is there someone available to help you with daily chores?”, “Can you count on anyone to provide you with emotional support?”, and “Do you have a much contact as you would like with someone you feel close to, someone in whom you can trust and confide?”). Answer options were reversed: 1 = none of the time, 2 = a little of the time, 3 = some of the time, 4 = most of the time, and 5 = all the time. Summed scores ranged from 7 to 30, with higher scores indicating higher levels of social support. The measure demonstrated good reliability in this sample (Cronbach’s α = 0.856; 95% CI: 0.817, 0.885).

Physical problems severity (Short Form-8, SF-8). Four items assessed physical health during the past four weeks (i.e., “During the past 4 weeks, how much did physical health problems limit your physical activities?”, “During the past 4 weeks, how much difficulty did you have doing your daily work, both at home and away from home because of your physical health?”, “How much bodily pain have you had during the past 4 weeks?”, and “During the past 4 weeks, how much did your physical health or emotional problems limit your usual social activities with family and friends?”). For items on limitations on physical activities, answer options were 1 = not at all, 2 = very little, 3 = somewhat, 4 = quite a lot, and 5 = could not do daily work. For items on bodily pain, answer options were 1 = none, 2 = very mild, 3 = mild, 4 = moderate, 5 = severe, and 6 = very severe. Summed scores ranged from 4 to 21, with higher scores representing more physical problems severity. The measure demonstrated good reliability in this sample (Cronbach’s α = 0.848; 95% CI: 0.806, 0.879).

Anxiety symptoms. It was measured using one item (i.e., “Do you have the following health problem? Anxiety”). Response categories scored 0 = no, and 1 = yes.

Sociodemographic characteristics. Sex was a binary variable with values 0 = male and 1 = female. Age was a continuous variable grouped in three categories: 0 = 18–44, 1 = 45–64, 2 =≥ 65. Education (years) was a continuous variable grouped as 0 =< 12 and 1 =≥ 12. Time living in the US was a continuous variable grouped in three categories: 0 = born in the US, 1 = born in Mexico and ≤30 years living in the US, and 2 = born in Mexico and >30 years living in the US (the cut-off points for this variable aimed to distribute the sample more equitably). Finally, county was a dummy variable with values 0 = Pima, 1 = Yuma, and 2 = Santa Cruz.

### 2.5. Data Analysis

This study pre-determined the one-factor structure (confirmatory factor analysis (CFA)) and verified the psychometric structured of previously culturally adapted scales using the marker method (i.e., it responded how common variance was shared among the items using Lavaan package in R 4.1). Items 1–4, 6, and 9–10 of our depressive symptom scale were reliable estimate measures of depression; items 1–6 of the adapted ENRICHED and SHS scales were reliable estimate measures of social support and hope, respectively; and items 2–4, and 6 of the SF8 scale were reliable estimate measures of severe physical problems severity. Despite the fact the missing data were below 5% for all variables, we also utilized predictive mean matching to impute missing data (i.e., it randomly filled values from observed donor values using the Mice package in R 4.1); five imputations were combined in original observed data.

**Figure 1:**
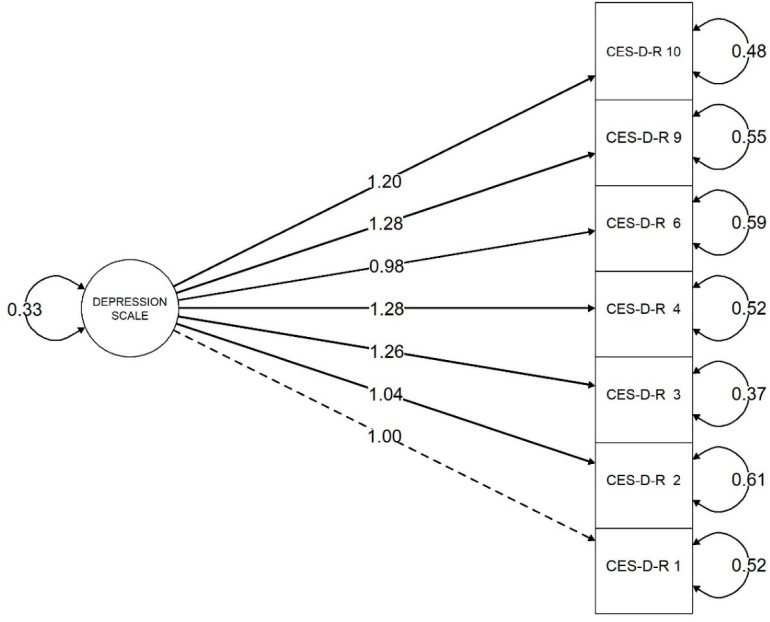
Diagram of the confirmatory factor analysis on depressive symptoms.

Figure 1 shows a path diagram on how much common variance is shared among the items. The circle represents the latent variable (i.e., depressive symptoms), the squares represent the observed indicators (i.e., CES-D-R 1, CES-D-R 2, CES-D-R 3, CES-D-R 4, CES-D-R 6, CES-D-R 9, and CES-D-R 10), the one-way arrows represent the paths, and the two-way arrow represents the variance/covariance. For instance, for one standard deviation in CES-D-R 10, item 10 goes up by 1.20 standard deviation units in the scale of item 1 (the residual of the variance of item 10 is 0.48, and the variance of the factor is 0.33 in the scale of item 1). Following conventions for good model fit [25], the model displays a Standardized Root Mean Square Residual (S-RMR) < 0.08 (0.045) and a Tucker–Lewis Index (TLI) > 0.9 (0.921).

The ordinary least square assumptions were tested for the variables (i.e., linearity, normality, multicollinearity, and homoscedasticity). Accordingly, seven outliers were dropped, and the depression, hope, physical problems severity, and social support scales ran as a logarithmic variables in the models. Bivariable and multivariable linear regression models were conducted to explore the determinants of the depressive symptoms from the past four weeks adjusting for sociodemographic characteristics. Interactions between study covariates with depressive symptoms were examined to determine whether any of the variables functioned as moderators (only one of them was statistically significant at *p*-value < 0.05 and was included in the final model).

## 3. Results

Table 1 summarizes the baseline characteristics of the analytic sample. Most participants were female (85.9%) and between 45 and 64 years of age (49%). Over half had up to 12 years of education (54.4%). Only 16% were born in the US, and 46.6% had lived in the US for at least 30 years. From the total sample, 26.8% and 27.2% reported having depressive symptoms and anxiety symptoms, respectively. On average, participants reported relatively low levels of depressive symptoms (11.5 in a range from 7 to 26) and physical problems severity (9.4 in a range from 4 to 21). They also reported high levels of hope (24.5 in a range from 11 to 30) and social support (23.9 in a range from 7 to 30).

Table 2 presents five ordinary least squares models with imputed data (Models 1, 2, 3, 4, and 5). Model 1 includes health-related emotional, physical, and social characteristics as explanatory variables. Model 2 displays sociodemographic characteristics. Model 3 exhibits health-related physical and social characteristics, as well as sociodemographic characteristics. Model 4 presents health-related emotional, physical, and social characteristics, as well as sociodemographic characteristics. This model assumes that the effect of the hope variable is constant across the categories of anxiety. That is, if there is a statistically significant association between hope and depression, this is so, whether respondents self-report anxiety symptoms or not. Model 5 shows that this assumption might be untenable. There is a marginally significant interaction between hope and anxiety. After controlling for all covariates, the final model (Model 5) shows that hope had a negative and significant (*p* < 0.05) main effect on participants’ depressive symptoms (β = −0.53; 95% CI = −0.78, −0.29). Anxiety symptoms might moderate the association between hope and depressive symptoms (β = −0.31; 95% CI = −0.65, 0.02), where the presence of anxiety symptoms marginally (*p* < 0.1) reduced the positive effect of hope on depressive symptoms. Physical problems severity was positively and significantly (*p* < 0.05) associated with respondents’ depressive symptoms (β = 0.22; 95% CI = 0.13, 0.30), where the presence of physical problems severity was related to higher levels of depressive symptoms. Social support, sex, age, education, place of birth and time living in the US, and location did not have significant main effects or interaction effects on depressive symptoms.

## 4. Discussion

This study used cross-sectional data among Mexican-origin adults with chronic disease risk from three border counties in Arizona to examine the correlation of depressive symptoms. The overall proportion of depressive symptoms was estimated as 26.8%, which is similar to previous reports of depressive symptoms among Latine and Mexican-origin adults in the US [7,26,27]. The statistical analysis revealed that the effect of anxiety symptoms on depressive symptoms might be informed by participants’ levels of hope (i.e., greater hope attenuated the effect of anxiety symptoms on depressive symptoms). Additionally, physical problems severity was positively associated with depressive symptoms. This study complements prior research by conducting confirmatory factor analysis for the main variables, including imputations for missing data, using multivariable models to examine explanatory factors, and examining incremental changes (vs. odds ratios).

Our study found an expected overlap in depressive symptoms and hope, and anxiety symptoms might moderate this association. However, anxiety symptoms were measured with a single item, rather than a scale capturing different dimensions of this construct. Thus, the marginally significant interaction effect must be interpreted with caution. Additionally, the project dataset did not distinguish by levels of anxiety symptoms; thus, it was not possible to conduct a closer analysis of the relationship between gradients of anxiety symptoms and depressive symptoms (e.g., high depressive symptoms/low anxiety symptoms, high anxiety symptoms/low depressive symptoms, high depressive symptoms/high anxiety symptoms, low depressive symptoms/low anxiety symptoms). Future research may consider other measures of anxiety symptoms (e.g., the 10-item State-Trait Anxiety Inventory) to conduct this kind of analysis. This study also did not collect information on whether participants were being treated for depressive symptoms. Future research may also contemplate exploring antidepressant medication use and other interventions among this population. A study showed that 5% of a cohort of Latine adults with similar overall prevalence of depressive symptoms used antidepressant medication [26]. This information may be helpful in developing effective strategies to self-manage depressive symptoms and anxiety symptoms among this priority population.

Hope and related constructs such as optimism may offer a form of resilience against depressive symptoms [11,13]. A study of Mexican-origin women living in Northern California identified life experiences related to hopelessness to include partner issues (e.g., fights, deceptions, ruptures, substance use, and incarceration), family issues (e.g., pregnancy, separation, illness, and death), feelings of being alone (e.g., physical separation from loved ones), the inability to provide for material needs (e.g., transportation and housing), and bodily symptoms and experiences (e.g., giving birth and menstruation) [12]. More qualitative research is needed to understand the relationships between anxiety symptoms, hope, and depressive symptoms among Mexican-origin adults in the US–Mexico border region.

Prior research showed a positive association between depressive symptoms and poor physical health among Latine individuals [27,28]. This study supports and complements those findings with a case study in the US–Mexico border region. Considering that Mexican-origin individuals may express depressive symptoms in terms of somatic complaints (vs. psychological or psychiatric complaints) [29], CHWs play a key role in validating their complaints by recognizing the importance of mental healthcare and by referring them to health services. Moreover, there is a need for culturally competent healthcare providers to improve depressive symptoms detection among this population [30]. Additionally, future research may contemplate including objective measures of physical health among this priority population, including the experience of pain. 

Social support can facilitate coping with depressive symptoms (e.g., familismo, personalismo, and respeto) [31]. Considering that Mexican culture often reinforces collectivism and affiliation, it is possible that Mexican-origin adults rely on interpersonal relationships as the primary source for coping with daily hassle, and feel vulnerable and undervalued when they lack social support [15]. Nonetheless, this study found no significant association between social support and depressive symptoms. Thus, future studies may consider disaggregating social support, such as the Social Support Inventory scale, to evaluate how each type of support (e.g., emotional, informational, instrumental, and appraisal) and form of delivery (e.g., direct vs. indirect) is related to depressive symptoms. Likewise, future studies on Mexican-origin adults may focus on social interactions (e.g., family, relatives, and friends) that are negative in nature (e.g., criticism and rejection) [32]. Prior research also found that perceived discrimination (e.g., being treated unfairly, disliked, disrespected, rejected, or stereotyped) and perceived stress predicted depressive symptoms among Mexican-origin males and females [33]. Thus, future studies may consider if discrimination and stress are related to depressive symptoms among Mexican-origin adults in the US–Mexico border region.

Depressive symptoms have been consistently more prevalent in females than males due to socioeconomic, biological, personality, stress, styles, and distress differences [10,13]. Though this study did not find such associations to be significant. Prior studies found that lower levels of SES and education were significantly related to a higher risk of major depressive symptoms in Latine adults in general [34]. However, in this analysis, education was not a significant predictor of depressive symptoms among Mexican-origin individuals. Previous studies also found mixed evidence on the association between depressive symptoms and being born in the US or time living in the US [10,26]. Nonetheless, this study did not find this association to be significant.

### Limitations

These data derived from the LINKs study are characterized by strengths and limitations. Among the former are its focus on a high-need population in an underserved area, and its CCL model, in which CHWs connect participants to health and social services, as well as broad and regular engagement with community organizations to inform all aspects of this research. Among the latter are the homogeneous target population and sample. These findings represent mainly Mexican-origin adult females who attend community health clinics in Yuma, Pima, and Santa Cruz, Arizona. Thus, future research may consider including more males, as well as comparing Mexican-origin and non-Mexican-origin individuals who attend community health clinics and those who receive healthcare elsewhere, or not at all, within the US–Mexico border region. Additionally, research shows that assessing both subjective and objective physical health may provide additional explanatory power in predicting depressive symptoms among Mexican-origin adults [7]. However, this analysis relied only on subjective indicators of health not accounting for chronicity and number of episodes. Future research could compare both objective and subjective types of physical health. Considering only one item to measure anxiety, this analysis did not distinguish between anxiety disorders and symptoms of anxiety. Future studies should incorporate independent instruments/scales to assess both anxiety disorder and anxiety symptoms. Likewise, future studies may consider controlling for other factors that contribute to depressive symptoms, such as medication use, history of mental health, and other life stressors. In the context of the US–Mexico border region, it is also necessary to include more measurements of the structural determinants of health. For instance, how social stressors (e.g., perceived discrimination and repeated exposure to discrimination) may directly or indirectly affects depressive symptoms (e.g., psychological and physiological responses). Due to the specific sample and cross-sectional nature of this study, it was not possible to establish the generalizability of findings or determine temporal relationships in the conducted analysis. Finally, Model 5 showed a marginally significant interaction effect (*p*-value = 0.1) of anxiety symptoms and hope on depressive symptoms. Thus, future research may consider studying this association in more depth (e.g., longitudinal analysis).

## 5. Conclusions

Despite Mexican-origin adults being overrepresented among low-income and undeserved groups in the US, there is a significant gap between their need for and availability of health services. This study looked at the psychosocial, demographic, and geographic correlates of depressive symptoms among Mexican-origin adults residing in three Arizona border counties. The main findings revealed a high overall proportion of depressive symptoms (26.8%) in our sample, where physical problems severity was positively related to depressive symptoms, and the effect of anxiety symptoms on depressive symptoms might be informed by participants’ levels of hope. These findings may help direct community service and local health department efforts to provide better information to plan effective prevention efforts intended to achieve health equity and to eliminate health disparities. The collection and diffusion of specific knowledge on depressive symptoms among Mexican-origin adults are relevant, considering that they may prefer self-care, counseling, and support from members of their social network over medication and healthcare from mental health specialists [5,35]. Moreover, the more individuals know about depression and its symptoms, the less likely they are to experience stigma about accessing mental healthcare services, and the more likely they are to engage with a person who has been treated for depressive symptoms [36]. Considering the US–Mexico border region’s distinct overall culture, lack of healthcare access, historical exclusion of foreign-born people in vital safety net programs, and stigma associated with mental health help-seeking [9], understanding the context of the mental health problems of its residents to better inform the potential role for CHWs in identifying and navigating health services is an important public health goal. CHWs may be in a unique position to provide support for people with depressive symptoms (e.g., health education, preventive health screening, chronic disease prevention and management, navigation of health/social systems, community capacity building, and community advocacy). Mexican-origin adults lacking hope, experiencing anxiety symptoms, and with severe physical problems are a priority population in southern Arizona in need of depressive-symptoms-related prevention and treatment approaches. Addressing physical pain and maximizing hope, CHWs have the opportunity to ameliorate depression among Latine adults in the US–Mexico border region.

## Figures and Tables

**Table 1:** Baseline characteristics of LINKS sample.

Variables	Overall (N = 206)
Depressive symptoms	
Mean (SD)	11.5 (4.31)
Median [Min, Max]	10.0 [7.0, 26.0]
Missing	9 (4.4%)
No	151 (73.2%)
Yes	55 (26.8%)
Anxiety symptoms	
No	150 (72.8%)
Yes	56 (27.2%)
Hope	
Mean (SD)	24.5 (4.82)
Median [Min, Max]	25.0 [11.0, 30.0]
Missing	8 (3.9%)
Physical problems severity	
Mean (SD)	9.35 (4.42)
Median [Min, Max]	9.00 [4.0, 21.0]
Missing	5 (2.4%)
Social support	
Mean (SD)	23.9 (5.63)
Median [Min, Max]	25.0 [7.0, 30.0]
Missing	3 (1.5%)
Sex	
Male	29 (14.1%)
Female	177 (85.9%)
Age (years)	
18–44	37 (18.0%)
45–64	101 (49.0%)
>=65	67 (32.5%)
Missing	1 (0.5%)
Education (years)	
<12	112 (54.4%)
>=12	86 (41.7%)
Missing	8 (3.9%)
Place of birth & Time in the US (years)	
US Birth	33 (16.0%)
MX Birth & US <= 30	96 (46.6%)
MX Birth & US >30	70 (34.0%)
Missing	7 (3.4%)
County	
Pima	93 (45.1%)
Yuma	65 (31.6%)
Santa Cruz	48 (23.3%)

**Table 2:** Multivariable linear regression models of the determinants of depressive symptoms over past 4 weeks among Mexican-origin adults in Pima, Yuma, and Santa Cruz, Arizona.

Variables	Model 1	Model 2	Model 3	Model 4	Model 5
Anxiety (Yes)	0.12 **			0.12 *	1.11 *
	[0.04, 0.21]			[0.03, 0.21]	[0.07, 2.15]
Hope	−0.66 ***		−0.68 ***	−0.64 ***	−0.53 ***
	[−0.84, −0.47]		[−0.88, −0.48]	[−0.84, −0.44]	[−0.78, −0.29]
Physical problems severity	0.22 ***		0.24 ***	0.22 ***	0.22 ***
	[0.13, 0.30]		[0.16, 0.32]	[0.13, 0.30]	[0.13, 0.30]
Social support	−0.09		−0.12	−0.09	−0.08
	[−0.22, 0.05]		[−0.26, 0.03]	[−0.23, 0.05]	[−0.22, 0.06]
Sex (Female)		0.02	−0.03	−0.05	−0.04
		[−0.12, 0.16]	[−0.14, 0.07]	[−0.16, 0.06]	[−0.15, 0.07]
Age (45–64 years)		0.03	0.01	0.00	0.00
		[−0.11, 0.16]	[−0.09, 0.12]	[−0.10, 0.11]	[−0.10, 0.10]
Age (>=65 years)		−0.16 +	−0.09	−0.09	−0.09
		[−0.31, 0.00]	[−0.21, 0.03]	[−0.21, 0.03]	[−0.21, 0.03]
Education (>=12 years)		−0.10 *	0.01	0.01	0.00
		[−0.20, −0.01]	[−0.06, 0.09]	[−0.07, 0.08]	[−0.07, 0.08]
MX birth and US <= 30 years		−0.06	0.00	0.00	−0.01
		[−0.21, 0.10]	[−0.12, 0.13]	[−0.12, 0.12]	[−0.13, 0.11]
MX birth and US > 30 years		0.08	0.02	0.02	0.01
		[−0.08, 0.24]	[−0.11, 0.14]	[−0.10, 0.14]	[−0.11, 0.14]
County (Yuma)		0.12 *	0.03	0.01	0.01
		[0.01, 0.23]	[−0.06, 0.12]	[−0.07, 0.10]	[−0.08, 0.09]
County (Santa Cruz)		0.10	0.05	0.05	0.03
		[−0.03, 0.24]	[−0.06, 0.16]	[−0.06, 0.15]	[−0.07, 0.14]
Anxiety (Yes):Hope					−0.31 +
					[−0.65, 0.02]
# Observations	206	206	206	206	206
# Imputations	5	5	5	5	5
R^2^	0.488	0.105	0.482	0.504	0.513
R^2^ Adj.	0.477	0.068	0.453	0.473	0.480

+ *p* < 0.1, * *p* < 0.05, ** *p* < 0.01, *** *p* < 0.001. The outcome variable, depressive symptoms, was logged in all ordinary least squares models. The five models included imputed data. US = United States; MX = Mexico.

## Data Availability

The data presented in this study are available on request from the corresponding author.

## References

[1] De Oliveira G., Cianelli R., Gattamorta K., Kowalski N., Peragallo N. (2017). Social Determinants of Depression Among Hispanic Women. J. Am. Psychiatr. Nurses Assoc..

[2] (2013). APA Depressive Disorders. Diagnostic and Statistical Manual of Mental Disorders: DSM-5.

[3] Kendzor D.E., Chen M., Reininger B.M., Businelle M.S., Stewart D.W., Fisher-Hoch S.P., Rentfro A.R., Wetter D.W., McCormick J.B. (2014). The association of depression and anxiety with glycemic control among Mexican Americans with diabetes living near the U.S.-Mexico border. BMC Public Health.

[4] Hooker K., Phibbs S., Irvin V.L., Mendez-Luck C.A., Doan L.N., Li T., Turner S., Choun S. (2019). Depression among Older Adults in the United States by Disaggregated Race and Ethnicity. Gerontologist.

[5] Provencio-Vasquez E., Mata H.J., Tomaka J., De Santis J.P. (2017). Depression, Self-Esteem, and Childhood Abuse Among Hispanic Men Residing in the U.S.–Mexico Border Region. J. Assoc. Nurses AIDS Care.

[6] Hinton L., Apesoa-Varano E.C., González H.M., Aguilar-Gaxiola S., Dwight-Johnson M., Barker J.C., Tran C., Zuniga R., Unützer J. (2012). Falling through the cracks: Gaps in depression treatment among older Mexican-origin and white men. Int. J. Geriatr. Psychiatry.

[7] Olvera R.L., Williamson D.E., Fisher-Hoch S.P., Vatcheva K.P., McCormick J.B. (2015). Depression, Obesity, and Metabolic Syndrome: Prevalence and Risks of Comorbidity in a Population-Based Representative Sample of Mexican Americans. J. Clin. Psychiatry.

[8] Garcini L.M., Chen M.A., Brown R., LeRoy A.S., Cano M.A., Peek K., Fagundes C. (2020). “Abrazame Que Ayuda” (Hug Me, It Helps): Social Support and the Effect of Perceived Discrimination on Depression among US- and Foreign-Born Latinxs in the USA. J. Racial Ethn. Heal. Disparities.

[9] Keeney A.J., Quandt A., Meng Y., Flores L., Flores D., Garratt R., Hernandez P., Villaseñor M. (2022). “We All Have a Job to Do in This World, It’s up to Us”: Farmworker and Farmer Mental Health in a Rural US-Mexico Border Region. J. Agromedicine.

[10] Gonzalez P., Gonzalez G.M. (2008). Acculturation, Optimism and Relatively Fewer Symptoms Among Mexican Immigrants and Mexican Americans. Psychol. Rep..

[11] Marsiglia F.F., Kulis S., Perez H.G., Bermudez-Parsai M. (2011). Hopelessness, Family Stress, and Depression among Mexican-Heritage Mothers in the Southwest. Health Soc. Work.

[12] Heilemann M.S.V., Coffey-Love M., Frutos L. (2004). Perceived reasons for depression among low income women of Mexican descent. Arch. Psychiatry Nurs..

[13] Myers H.F., Lesser I., Rodriguez N., Mira C.B., Hwang W.C., Camp C., Anderson D., Erickson L., Wohl M. (2002). Ethnic differences in clinical presentation of depression in adult women. Cult. Divers. Ethn. Minor. Psychol..

[14] Ryan D., Tornberg-Belanger S.N., Perez G., Maurer S., Price C., Rao D., Chan K.C.G., Ornelas I.J. (2021). Stress, social support and their relationship to depression and anxiety among Latina immigrant women. J. Psychosom. Res..

[15] Hovey J.D., Magaña C. (2000). Acculturative Stress, Anxiety, and Depression among Mexican Immigrant Farmworkers in the Midwest United States. J. Immigr. Health.

[16] Hernandez A., Sachs-Ericsson N. (2006). Ethnic differences in pain reports and the moderating role of depression in a community sample of hispanic and caucasian participants with serious health problems. Psychosom. Med..

[17] Dilsaver S.C., Benazzi F., Manning J.S., Akiskal K.K., Akiskal H.S. (2008). Pain complaints in Latino adults of Mexican origin with and without major depressive episode: A cross-sectional study. Prim. Care Companion J. Clin. Psychiatry.

[18] Craske M.G., Rauch S.L., Ursano R., Prenoveau J., Pine D.S., Zinbarg R.E. (2009). What is an anxiety disorder?. Depress. Anxiety.

[19] Jill Fleuriet K., Sunil T.S. (2014). Perceived social stress, pregnancy-related anxiety, depression and subjective social status among pregnant Mexican and Mexican American Women in South Texas. J. Health Care Poor Underserved.

[20] Lohr A.M., Ingram M., Carvajal S.C., Doubleday K., Aceves B., Espinoza C., Redondo F., Coronado G., David C., Bell M.L. (2019). Protocol for LINKS (linking individual needs to community and clinical services): A prospective matched observational study of a community health worker community clinical linkage intervention on the U.S.-Mexico border. BMC Public Health.

[21] Snyder C.R., Sympson S.C., Ybasco F.C., Borders T.F., Babyak M.A., Higgins R.L. (1996). Development and Validation of the State Hope Scale. J. Pers. Soc. Psychol..

[22] Casanova M.P., Nelson M.C., Pickering M.A., Larkins L.W., Appleby K.M., Grindley E.J., Baker R.T. (2021). Disablement in the Physically Active Scale Short Form-8: Psychometric evaluation. BMC Sports Sci. Med. Rehabil..

[23] Timmerman I.G.H., Emanuels-Zuurveen E.S., Emmelkamp P.M.G. (2000). The Social Support Inventory (SSI): A Brief Scale to Assess Perceived Adequacy of Social Support. Clin. Psychol. Psychother..

[24] Salk R.H., Hyde J.S., Abramson L.Y. (2017). Gender Differences in Depression in Representative National Samples: Meta-Analyses of Diagnoses and Symptoms. Psychol. Bull..

[25] Humble S., Humble S. (2020). Factor analysis: Confirmatory. Quantitative Analysis of Questionnaires: Techniques to Explore Structures and Relationships.

[26] Wassertheil-Smoller S., Arredondo E.M., Cai J.W., Castaneda S.F., Choca J.P., Gallo L.C., Jung M., LaVange L.M., Lee-Rey E.T., Mosley T. (2014). Depression, anxiety, antidepressant use, and cardiovascular disease among Hispanic men and women of different national backgrounds: Results from the Hispanic Community Health Study/Study of Latinos. Ann. Epidemiol..

[27] Mier N., Bocanegra-Alonso A., Zhan D., Wang S., Stoltz S.M., Acosta-Gonzalez R.I., Zuniga M.A. (2008). Clinical depressive symptoms and diabetes in a binational border population. J. Am. Board Fam. Med..

[28] Ranney M.J., Aranda M.P. (2001). Factors associated with depressive symptoms among latino family dementia caregivers. J. Ethn. Cult. Divers. Soc. Work.

[29] Organista K., Iwamasa G., Hays P., Hays P., Iwasama G. (2006). Cognitive-Behavioral Therapy with Latinos and Latinas. Culturally Responsive Cognitive-Behavioral Therapy: Assessment, Practice, and Supervision.

[30] Coulter K., Ingram M., Lohr A.M., Bell M.L., Carvajal S. (2021). Examining associations between community health worker-rated health and mental health among Latino adults with chronic disease. Int. J. Environ. Res. Public Health.

[31] Ingram M., Coulter K., Doubleday K., Espinoza C., Redondo F., Wilkinson-Lee A.M., Lohr A.M., Carvajal S.C. (2021). An integrated mixed methods approach to clarifying delivery, receipt and potential benefits of CHW-facilitated social support in a health promotion intervention. BMC Health Serv. Res..

[32] Viruell-Fuentes E.A., Andrade F.C.D. (2016). Testing Immigrant Social Ties Explanations for Latino Health Paradoxes: The Case of Social Support and Depression Symptoms. J. Latino/Latin Am. Stud..

[33] Flores E., Tschann J.M., Dimas J.M., Bachen E.A., Pasch L.A., De Groat C.L. (2008). Perceived discrimination, perceived stress, and mental and physical health among Mexican-origin adults. Hisp. J. Behav. Sci..

[34] Bromberger J.T., Harlow S., Avis N., Kravitz H.M., Cordal A. (2004). Racial/ethnic differences in the prevalence of depressive symptoms among middle-aged women: The Study of Women’s Health Across the Nation (SWAN). Am. J. Public Health.

[35] Edelblute H.B., Clark S., Mann L., McKenney K.M., Bischof J.J., Kistler C. (2014). Promotoras across the border: A pilot study addressing depression in Mexican women impacted by migration. J. Immigr. Minor. Health.

[36] Lopez V., Sanchez K., Killian M.O., Eghaneyan B.H. (2018). Depression screening and education: An examination of mental health literacy and stigma in a sample of Hispanic women. BMC Public Health.

